# Implementing a Novel Statewide Network to Support Emergency Department-initiated Buprenorphine Treatment

**DOI:** 10.5811/westjem.2022.3.54680

**Published:** 2022-06-05

**Authors:** Brian M. Clemency, Rachel A. Hoopsick, Susan J. Burnett, Linda S. Kahn, Joshua J. Lynch

**Affiliations:** * The University at Buffalo, Department of Emergency Medicine, Buffalo, New York; † The University at Buffalo, Department of Family Medicine, Buffalo, New York; ‡ University of Illinois Urbana-Champaign, Department of Kinesiology and Community Health, Urbana, Illinois

## Abstract

**Introduction:**

Medications for opioid use disorder (MOUD), including buprenorphine, represent an evidence-based treatment that supports long-term recovery and reduces risk of overdose death. Patients in crisis from opioid use disorder (OUD) often seek care from emergency departments (ED). The New York Medication for Addiction Treatment and Electronic Referrals (MATTERS) network is designed to support ED-initiated buprenorphine and urgent referrals to long-term care for patients suffering from OUD.

**Methods:**

Using the PRECEDE-PROCEED implementation science framework, we provide an overview of the creation of the MATTERS network in Western New York. We also include an explanation of how the network was designed and launched as a response to the opioid epidemic. Finally, we analyzed the program’s outputs and outcomes, thus far, as it continues to grow across the state.

**Results:**

The New York MATTERS network was created and implemented in 2019 with a single hospital referring patients with OUD to three local clinics. In the social assessment and situational analysis phase, we describe the opioid epidemic and available resources in the region at the outset of the program. In the epidemiological assessment phase, we quantify the epidemic on the state and regional levels. In the educational and ecological assessment, we review local ED practices and resources. In the administrative and policy assessment and intervention alignment phase, the program’s unique framework is reviewed. In the piloting phase, we describe the initial deployment of New York MATTERS. Finally, in the process evaluation phase, we depict the early lessons we learned. By the beginning of 2021, the New York MATTERS network included 35 hospitals that refer to 47 clinics throughout New York State.

**Conclusion:**

The New York MATTERS network provides a structured approach to reduce barriers to ED-initiated buprenorphine and urgent referral to long-term care. An implementation framework provides a structured means of evaluating this best practice model.

## INTRODUCTION

Opioid analgesic prescribing in the United States increased through the early 2000s, resulting in considerable increases in emergency department (ED) visits, inpatient hospitalizations, and overdose deaths.^1^–^5^ Nationally representative data from the National Hospital Ambulatory Medical Care Survey shows that the ED visit rate for opioid overdose increased fourfold from 1993 to 2010.^3^ Despite a national effort to control the epidemic of non-medical use of opioid analgesics and a plateauing of opioid analgesic-related ED visits^6^ and mortality rates^7^ in the early 2010s, the prevalence of heroin use rose dramatically during the same time period, with over 620,000 Americans reporting the use of heroin in 2011.^8^ The recent addition of fentanyl to the supply of heroin and other street drugs has resulted in a further increase in the risk of opioid overdose death^9^,^10^ observed across men and women and various racial/ethnic groups.^11^ Recent data shows that patients who are treated in the ED for non-fatal opioid overdose are at elevated risk for post-discharge mortality.^12^,^13^

Given that patients with opioid use disorder (OUD) frequently present to hospital EDs for acute opioid overdoses,^12^,^13^ opioid withdrawal symptoms,^14^ opioid-related infections,^5^ and concomitant psychiatric emergencies,^9^ these visits represent a critical opportunity for intervention to reduce opioid-related morbidity and mortality. Few hospitals have dedicated inpatient units for treatment of OUD; so most patients presenting at EDs with acute opioid overdose or withdrawal symptoms are stabilized and treated with non-narcotic symptom-relieving medications before being discharged.^15^,^16^ While some EDs have been able to leverage a harm reduction approach by providing take-home naloxone,^17^ this strategy has not shown long-term mitigation of overdose risk and subsequent ED presentation.^18^

Medications for opioid use disorder (MOUD), including buprenorphine, represent an evidence-based treatment for OUD that supports long-term recovery and reduced risk of overdose death.^19^ Given that ED visits represent a critical and time-sensitive point for patients with OUD, there has been a call for EDs to engage these patients with buprenorphine as a means of life-saving treatment.^20^ There is a growing body of literature demonstrating the feasibility and effectiveness of ED-administered buprenorphine.^14^,^21^–^24^

In emergency settings, patients who receive buprenorphine for opioid withdrawal are significantly more likely to be receiving MOUD one month after ED discharge than patients treated in the ED with clonidine, a non-narcotic, symptom-relieving medication.^25^ However, many of the studies demonstrating the feasibility of ED-administered buprenorphine refer these patients to a single hospital-affiliated clinic for ongoing MOUD, which is not a universally replicable model. Further, these pilot interventions do not address issues related to patients’ ability to afford MOUD after leaving the ED, which is important given that OUD is more prevalent among people who are uninsured, unemployed, and/or living in poverty.^26^ This necessitates a broader network of hospitals and community-based MOUD clinics and consideration of the financial burden of MOUD in addressing the opioid epidemic via ED-initiated buprenorphine.

Given the promise of ED-initiated buprenorphine as a public health approach to addressing the opioid epidemic^20^ and the need for a more scalable intervention model, the current study examines New York MATTERS (Medication for Addiction Treatment and Electronic Referrals), a novel statewide network developed as a public health response to the opioid epidemic that operates through ED-initiated buprenorphine treatment and linkages to community-based care. This paper describes the assessment, implementation, and evaluation tasks completed to date of New York MATTERS within the context of PRECEDE-PROCEED,^27^ a public health model for intervention planning and evaluation. This study was reviewed and approved by the Institutional Review Board of the University at Buffalo.

*Population Health Research Capsule*

What do we already know about this issue?

*Patients with opioid use disorder (OUD) often turn to an ED in times of crisis. Buprenorphine has demonstrated efficacy for the treatment of OUD.*

What was the research question?

*Using the PRECEDE-PROCEED framework, we describe the creation of a network to support ED-initiated buprenorphine treatment.*

What was the major finding of the study?

*We demonstrate that the establishment of a large-scale network to support ED-initiated buprenorphine and referrals for ongoing care is feasible.*

How does this improve population health?

*The Medication for Addiction Treatment and Electronic Referrals network represents a best practice model for ED-initiated buprenorphine and referrals for ongoing OUD care.*

## METHODS

New York MATTERS originated in late 2017 in Erie County. Located in western New York State, it is the ninth most densely populated of the state’s 62 counties. It is a racially and ethnically diverse county that includes Buffalo, the second largest city in the state. The New York MATTERS network originated in a university-affiliated teaching hospital located in Buffalo, NY.

**Figure 1:**
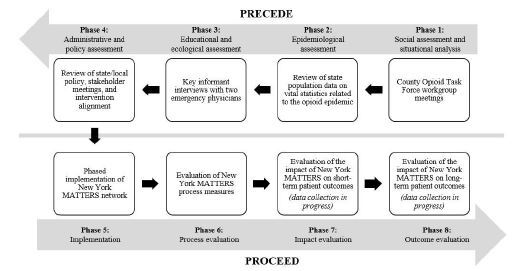
Adapted PRECEDE-PROCEED framework. *MATTERS*, Medication Treatment and Electronic Referrals network.

PRECEDE-PROCEED^27^ is a framework used for health promotion planning and evaluation. PRECEDE is an acronym that stands for “*predisposing, reinforcing, and enabling constructs in educational/ecological diagnosis and evaluation*”^27^ and represents a series of assessments that generate information used to guide subsequent decisions in the design and implementation of the intervention. The first four phases of the model represented by PRECEDE are as follows: 1) social assessment and situational analysis; 2) epidemiological assessment; 3) educational and ecological assessment; and 4) administrative and policy assessment and intervention alignment. PROCEED, the second half of the model, stands for “*policy, regulatory, and organizational constructs in educational and environmental development.*”^27^ Phases 5–8 represented by PROCEED include the following: 5) implementation; 6) process evaluation; 7) impact evaluation; and 8) outcome evaluation. Using a rapid implementation science approach, we completed phases 1–6 of PRECEDE-PROCEED and report the results here. Data regarding the effects of the New York MATTERS network intervention on patients’ health and quality of life is still being collected. Thus, we excluded data for Phases 7 and 8 of PRECEDE-PROCEED from the current study. An adapted PRECEDE-PROCEED framework that informed this intervention is shown in Figure 1.

### Phase 1: Social Assessment and Situational Analysis

In response to the rapidly evolving opioid epidemic, Erie County declared a public health crisis in 2016 and formed an Opioid Epidemic Task Force through executive order. This task force included members of the community (including those with and impacted by OUD), emergency physicians, outpatient community-based MOUD physicians, members of the Erie County Department of Health, and representatives from regional health insurers, as well as members of local law enforcement and the District Attorney’s Office who deal with drug-related criminal justice issues. Through observation of monthly workgroup meetings, the challenges and priorities of people with OUD living in Erie County were subjectively defined.

### Phase 2: Epidemiological Assessment

We accessed aggregated state population data^28^ to understand vital indicators of the opioid epidemic in Erie County, NY. Indicators included opioid-related mortality rates, opioid-related ED visits and hospitalizations, and opioid-related treatment entries in community-based settings. These data were used to rank and prioritize problems related to the issues identified in Phase 1.

### Phase 3: Educational and Ecological Assessment

Following our epidemiological assessment of the opioid crisis in Erie County, we explored the educational and ecological needs of emergency physicians. We informally conducted key informant interviews with two emergency physicians at a single teaching hospital and identified predisposing, enabling, and reinforcing factors^27^ to implementing ED-initiated buprenorphine and establishing linkages to community-based clinicians. One physician interviewed is the director of emergency medicine (EM) at a teaching hospital in Erie County and is board certified in EM. This senior physician practices EM at two community hospitals, serves as a technical advisor for the state and local departments of health, and notably serves on an advisory panel of the state’s Office of Addiction Services and Supports. This physician also engages in prehospital care by serving as medical director for an emergency air medical transport service and several fire and police departments in the region. The second physician interviewed is board certified in EM and practices at three hospitals in Erie County. This physician also serves as the medical director for several fire departments in the region.

### Phase 4: Administrative and Policy Assessment and Intervention Alignment

We matched our strategies and intervention components with the desired outcomes identified in Phase 1 and Phase 2 and determined whether the capabilities and resources were available to develop and implement the program.^27^ Through a review of state and local policy data and multiple meetings with the Opioid Epidemic Task Force, we determined the feasibility of the New York MATTERS network and created a list of intervention components and their alignment to identify predisposing, enabling, and reinforcing factors is presented.

### Phase 5: Implementation

Using resources identified in Phase 4 and aligned intervention components, the New York MATTERS network began a phased approach to implementation in 2017. We present a timeline of the New York MATTERS implementation network and describe the phased implementation approach.

### Phase 6: Process Evaluation

Following the initial launch of the New York MATTERS network, a continuous quality improvement process began. A trained program coordinator maintained a database of process outcomes that included the following: the number of participating hospitals and other referral sites; the number of participating community-based MOUD clinics; the number of participating pharmacies; the number of emergency physicians, nurse practitioners, and physician assistants (collectively referred to as EM clinicians) trained in buprenorphine prescribing; and the number of patients served. Additionally, necessary modifications to the intervention were identified and made during the scale-up process of the phased implementation and are described.

## RESULTS AND DISCUSSION

### PHASE 1: Social Assessment

Following the formation of the Opioid Epidemic Task Force, the challenges and priorities of people with OUD living in Erie County were defined and committees were formed to address each identified problem area. This process resulted in the formation of a “Hospital ER Committee” to focus on hospital access to MOUD as a public health approach to treating patients with OUD who present to the ED. This committee was led by an emergency physician and included representatives from all major hospital systems in the region, emergency medical services (EMS) agencies, and counselors from the county’s 24/7 addiction hotline. These committees each meet approximately once per month, and the full Opioid Epidemic Task Force meets quarterly where committees report on progress. The formation of the Hospital ER Committee brought together leaders from local hospitals with a focus on this goal and allowed them to leverage the expertise of other stakeholders from the larger task force.

### PHASE 2: Epidemiological Assessment

Aggregated population data^28^ documented a significant increase in opioid-related mortality in New York State; the age-adjusted rate of opioid overdose deaths increased nearly threefold from 5.4 per 100,000 population in 2010 15.1 per 100,000 population in 2016, with the greatest increases in heroin (1.0 to 6.5 per 100,000 population in 2010 and 2016, respectively) and synthetic opioid overdose (0.9 to 8.3 per 100,000 population in 2010 and 2016, respectively) death rates. County-specific data revealed that Erie County had the highest number of opioid overdose deaths in 2016 (N = 274), second only to Suffolk County (N = 344), which has a much larger population. In 2016, Erie County also had an opioid overdose mortality rate that was more than twice that of the state as a whole, at 31.3 per 100,000 population.

Data shows that the crude rate of opioid overdose ED visits in 2016 was 56.9 per 100,000 population across New York State but was 139.8 per 100,000 in Erie County that same year, higher than any other county in the state. However, New York State had a crude rate of opioid-related hospital discharges of 130.2 per 100,000 population in 2016, while Erie County’s rate of opioid-related hospital discharges was only 90.2 per 100,000 population. This suggested that the greatest hospital burden of opioid overdose was occurring in the ED setting in Erie County, whereas other counties may have had more dedicated inpatient units for the treatment of OUD.

Despite having some of the highest rates of opioid overdose mortality and ED visits in New York State, Erie County’s rate of patient admissions into a state-certified outpatient treatment program in 2017 was 474.8 per 100,000 population, falling below that of 30 other counties in the state. This suggested that there was unrealized potential to address the opioid epidemic by facilitating linkages from the ED to outpatient treatment programs in Erie County. This data was discussed and supported the notion of expanding access to MOUD in ED settings and establishing a network to enable patients to link to outpatient programs for continued MOUD. The data was also useful in demonstrating the extent of these challenges to hospital leaders outside of the EDs as well as potential external funders.

### Phase 3: Educational and Ecological Assessment

The two emergency physicians interviewed as key informants identified several predisposing, enabling, and reinforcing factors^27^ to implementing ED-initiated buprenorphine and establishing linkages to community-based MOUD clinics **(**Table 1). These findings are important because they illuminate barriers and facilitators to implementing this type of intervention that may be common to other communities affected by the opioid crisis.

#### Predisposing Factors

##### Clinician self-efficacy to appropriately determine buprenorphine dosing

The appropriate, patient-specific, starting buprenorphine dose is typically determined through an assessment of the patient’s opioid use patterns and risk for withdrawal. The dose is then slowly titrated upward until withdrawal symptoms are satisfactorily abated. Clinicians expressed concern regarding their ability to properly determine the appropriate dose, and the time and resources needed in a busy ED to observe the patient during a gradual titration.

##### Clinician self-efficacy to provide patients with follow-up instructions

Once patients are discharged from the hospital, they require clear follow-up instructions. Clinicians admitted that they did not feel sufficiently knowledgeable to instruct patients on how to take buprenorphine at home (especially the first dose), how to obtain medication if they did not have health insurance, and how to navigate their first follow-up appointment. Literature suggests that clinicians are apprehensive about unobserved “home” buprenorphine induction due to the risk of diversion and precipitated withdrawal.^29^ However, this method has been shown to be feasible with low rates of adverse events.^30^

##### Clinician-perceived risk of buprenorphine diversion

Both licit and illicit opioids can be bought and sold on the street. Clinicians expressed concern that the medication they prescribed in the ED would be sold by patients or otherwise diverted. This is a common perception among buprenorphine prescribers in the United States.^31^

#### Enabling Factors

##### Clinician X-waiver to prescribe buprenorphine

In addition to a standard Drug Enforcement Administration (DEA) license, at the time of the rollout, physicians were required to complete an eight-hour training to obtain an “X-waiver” to prescribe buprenorphine. Nurse practitioners and physician assistants were required to complete 24 hours of training to obtain the waiver. Clinicians in the ED perceived this requirement as a barrier, in addition to the multiple other unfunded certification courses they must complete for licensure and hospital privileges.

##### Process for timing and location of clinician referral to community-based clinic

Based on the experiences shared by patients with OUD, clinicians worried that they would not be able to secure immediate follow-up appointments for their patients with a community-based clinician following an ED visit. In addition to the timing of a follow-up appointment, clinicians expressed concern about the multiple permutations necessary to determine the appropriate clinics to which they could refer patients. At the time, there were several community-based MOUD clinics in the region and each had unique rules regarding patient acceptance, such as health insurance coverage, concomitant benzodiazepine use, or a previous discharge because of failure to follow the clinic policies.

##### Process for scheduling follow-up appointment at community-based clinic

Given the heterogeneity of community-based clinics and scheduling processes, clinicians expressed a desire for a streamlined process to schedule patients’ follow-up appointments. Clinicians indicated that a streamlined process would reduce the time and administrative burden of linking patients to community-based care for continuing MOUD.

##### Patient ability to pay for buprenorphine

Many patients with OUD are uninsured or underinsured.^26^ Clinicians expressed concern about their patients’ ability to afford a prescription of buprenorphine outside a safety net setting and identified this as a practical barrier to implementing ED-initiated buprenorphine.

#### Reinforcing Factors

##### Extrinsic motivation to obtain/maintain X-waiver

Clinicians identified the need for some type of extrinsic motivation to reinforce ED clinicians’ securing and maintaining DEA X-waivers to prescribe buprenorphine. Research has shown that “pay-for-participation” incentives like honorariums are a viable strategy to motivate clinician behavior.^32^

### Phase 4: Administrative and Policy Assessment and Intervention Alignment

#### Standardized dosing scheme for clinicians

To improve ED clinicians’ self-efficacy with buprenorphine dosing, a standardized dosing scheme was created. The program first recommended an initial 4-milligram (mg) dose of buprenorphine followed by 4 mg of buprenorphine twice a day for three days. This recommended standardized dosing scheme was created by the physician leadership of the program in consultation with local addiction medicine specialists. Prescribers were still able to use a patient-specific dose and duration at their discretion, but most clinicians used the standardized scheme.

#### Standardized patient instructions for clinicians

To address ED clinicians’ self-efficacy to provide patients with follow-up instructions, standardized patient instructions were created as part of a packet that could easily be used by emergency clinicians. The packet included a decision-support flow chart, the phone number to the central referral line, and discharge instructions with blank spaces for patient-specific instructions, such as the date and location of their follow-up appointment. Not all patients with OUD presenting to EDs are appropriate for buprenorphine induction in the ED. For patients who were not induced in the ED, these instructions also included guidance on when to take their first dose of medication using non-technical language.

#### Diversion education for clinicians

To address concerns about buprenorphine prescription diversion by ED patients, ED clinicians were educated regarding the true buprenorphine diversion risk. Evidence shows that the majority of people who use illicit buprenorphine do so to manage opioid withdrawal symptoms or achieve or maintain abstinence from other opioids.^33^ Buprenorphine diversion is frequently a reflection of a persistent lack of treatment availability and barriers to buprenorphine access among people with OUD.^34^

#### Funded hybrid trainings for clinicians

To facilitate clinicians’ obtainment of a DEA X-waiver to prescribe buprenorphine, the Department of EM at the university affiliated with the hospital streamlined the waiver process to make participation more appealing. Leveraging a hybrid model, physicians first participated in four hours of online, asynchronous training and then attend a four-hour, face-to-face training. These regional trainings were funded by the university’s ED and were held at restaurants to foster a collegial environment and encourage clinician participation.

#### Mission, vision, and values document for clinic participation in network

To facilitate a process for timing and location of clinician referral to community-based clinics, a mission, vision, and values document was created to detail the goals of the network and the obligations of both ED clinicians and clinics receiving referrals through the network. A key component of this document was the obligation for clinics to accept all patients referred through the network. If a clinic could not meet the needs of a particular patient, it was the clinic’s responsibility to initiate care and then facilitate a secondary referral. Participating clinics included those that were single site, multisite, urban, suburban, and rural. Leadership from these clinics were asked to identify days of the week in which they would have the ability to accept a patient from the referral network to ensure that patients were seen within 24–72 hours after their ED visit. These were not dedicated appointments; rather they were periods in which the clinic felt their capacity was adequate to absorb an additional patient into the schedule on short notice. The referral process provided patients with the date and location of their first follow-up appointment. The clinic contacted the patient on the next business day to select a mutually agreeable appointment time.

#### Centralized referral system

To address clinicians’ desire for a streamlined process for scheduling a follow-up appointment at a community-based clinic, a centralized referral system was developed. A referral phone line with 24/7 availability was established by partnering with an EMS dispatch center. This enabled ED staff to call the hotline and instantly find clinic availability within the necessary referral period. Once an appropriate clinic appointment was selected, the dispatcher obtained patient information and sent it directly to the community-based clinic via a fax that was compliant with the Health Insurance Portability and Accountability Act (HIPAA). The dispatcher would also note that the scheduled clinic appointment was no longer available in the master log to avoid double-booking clinics.

#### Patient voucher for buprenorphine prescription

To address patients’ ability to pay for their buprenorphine prescription, New York MATTERS founders established a voucher program. This program provided patients’ initial outpatient buprenorphine prescription at no cost through the use of a voucher if they could not afford the medication. All uninsured patients and patients who reported that they were covered by Medicaid were automatically given a voucher for buprenorphine. If the patient arrived at the pharmacy and had active insurance, then the voucher was not charged. This was established through a partnership with a local pharmacy association that had experience with operationalizing voucher programs and had relationships with a network of local pharmacists who were willing to honor their vouchers. Patients identified as uninsured or underinsured received a voucher in the ED with information on participating pharmacies.

#### Clinician honorariums

To facilitate clinicians’ extrinsic motivation to obtain and maintain a DEA X-waiver to prescribe buprenorphine, the Department of EM at the university affiliated with the hospital where New York MATTERS originated provided $200 honorariums to clinicians once they completed the required training and obtained their DEA X-waiver. This was a temporary incentive offered only during the piloting and early phasing in of New York MATTERS. A summary of the intervention components is shown in Table 2.

### Phase 5: Implementation

#### Piloting

New York MATTERS originated in late 2017 and was piloted under the name “Buffalo MATTERS.” The initial launch was supported by a one-year, $200,000 grant from a community-based, not-for-profit organization. The pilot network consisted of one teaching community hospital (including two emergency physicians) and three community-based MOUD clinics. During this pilot stage, the clinician and patient worked together to complete a short, paper referral form with questions on patient contact information, medical history, past drug use, and mental health history. On the final page, the patient then ranked which clinic they would like to go to. The patient was also given buprenorphine discharge instructions. The clinician, unit secretary, or counselor called the centralized referral line with this information, and it was recorded on a master spreadsheet. Once the appointment slot was determined, patient information was relayed by the dispatcher to the appropriate clinic’s intake department, which then contacted the patient to confirm the exact appointment time.

#### Phasing In

**Figure 2:**
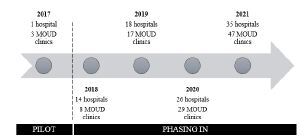
Timeline of implementation of the New York MATTERS* network. *MATTERS, Medication Treatment and Electronic Referrals network; *MOUD*, medications for opioid use disorder.

Over the next three and one-half years, New York MATTERS continued with a phased-in approach to implementation **(**Figure 2). New hospitals and community-based MOUD clinics joined the network after reviewing the mission, vision, and values document and agreeing to the obligations of both ED clinicians and clinics receiving referrals through the network. After clinic leadership agreed to these obligations, they completed a short, data collection form, which included information on clinic location, contact information, MOUD types prescribed (ie, buprenorphine, naltrexone, methadone), and appointment availability. Clinics provided appointment “slots,” as opposed to exact appointment times, given that the slot may or may not need to be used. For example, a clinic might offer two appointment slots every Monday and Wednesday where they agree to “squeeze in” a patient.

### Phase 6: Process Evaluation

Data collected by the program coordinator indicates that the network expanded from a small regional pilot to a statewide network of ED-initiated buprenorphine treatment and linkages to community-based care. To date, the New York MATTERS network includes 35 hospitals and a total of 47 community-based clinics across 83 individual sites (Table 3). Additionally, 22 pharmacies now partner with New York MATTERS and accept the network’s MOUD vouchers. A total of 228 ED clinicians have been trained by the network in buprenorphine prescribing and obtaining an X-waiver, and 394 patients with OUD have received ED-initiated buprenorphine and were referred to community-based care with a community-based network MOUD clinic. Further, leadership from an additional 12 hospitals and 14 community-based clinics have expressed an intent to join the network over the next year, expanding the size of New York MATTERS to an expected 49 hospitals and 61 community-based clinics by 2022.

The process evaluation also identified several modifications that needed to be made to the intervention. The centralized referral system was critical to the initial launch of New York MATTERS. However, the growing number of available clinics and the need to standardize the data collection process for referrals required a more robust information technology solution. Through a partnership with the New York State Department of Health, an online referral portal was created. Instead of a paper referral form, patients reviewed a growing list of clinics via a Wi-Fi-enabled tablet prior to their departure from the ED. After entering their information, patients were able to view a list of available clinics and dates from which to choose follow-up care. Patients could search for specific clinics, look for available sites near their homes, and select a site based on MOUD types available at that location.

Additionally, despite follow-up appointments being scheduled within 72 hours of discharge, we identified several instances when an appointment needed to be postponed or authorized prescribers were not available for the first clinic appointment. Feedback from patients and clinicians, as well as observed best practices in other areas, suggested that the initial dose of 4 mg of buprenorphine was insufficient for induction. Accordingly, the standardized dosing scheme was changed to 8 mg for induction and 4 mg twice a day for seven days for the initial prescription. This change provided an adequate “bridge” prescription for buprenorphine until patients could be seen for follow-up in a community-based setting for continued MOUD.

The referral phone line was invaluable to the initial launch of the program. As the program continued to grow, the volume and scope of the referral network necessitated a more robust referral solution. Partnering with the New York State Department of Health, a secure, HIPAA-complaint, scalable, online referral system (OLRS) was created. Participating EDs were supplied with iPads that could be used to access the OLRS. This OLRS allowed patients and clinicians to enter patient information, browse the available referral sites, confirm their referral date and location, and generate electronic discharge instructions. Referral information was sent directly to the receiving clinic, eliminating the need for a phone call in the ED. Additionally, the OLRS’s functionality allowed for facilitation of linkage to peers, electronic medication vouchers, and sending discharge instructions via email and text message. Feedback from clinicians and patients reflected that the OLRS was faster and more user-friendly than the original referral phone line model. The OLRS also allowed for more reliable data collection because the patients or hospital staff entered data directly into the system using a combination of defined and free-text fields.

## LIMITATIONS

When analyzed through the first six phases of the PRECEDE-PROCEED model, the New York MATTERS network displays many strengths for treating people with OUD with ED-initiated buprenorphine, but there are some limitations associated with this rollout. The pilot study and subsequent spread of the program took place in an area with already existing resources and infrastructure, such as clinics, pharmacies willing to participate in voucher programs, and call centers available to facilitate scheduling. These resources may not be available in all communities, which may slow, limit, or completely prevent local implementation. Clinicians’ perceptions of self-efficacy and concerns regarding buprenorphine prescription were based on self-reports, which could have been caused by a social desirability bias. Additionally, the pilot study system is known for progressive healthcare and public health programming; so the buy-in may have been greater, simply because of past experiences with novel program adoption or because the system had funding for DEA X-waivers, hybrid training, and incentives. Finally, the educational and ecological assessment included the views of two emergency physicians; including additional relevant stakeholders may have yielded more robust information.

## CONCLUSION

New York State’s Medication Treatment and Electronic Referrals network provides compelling evidence that ED-initiated buprenorphine as a public health approach to addressing the opioid epidemic^20^ can be expanded into a scalable intervention model, operating through a broad network of hospitals and community-based clinicians. This review provides evidence that the financial burden of providing medications for opioid use disorder can and should be addressed through these types of interventions. Data collection efforts regarding patients’ health and quality of life are ongoing. Further research is needed to examine the effects of the New York MATTERS network intervention on long-term outcomes for these patients with opioid use disorder who present to the ED. As the network expands into new regions, future study is needed to examine the scalability and the generalizability of this intervention.

## Figures and Tables

**Table 1:** Predisposing, enabling, and reinforcing factors for emergency clinicians to prescribe buprenorphine and refer patients to follow-up clinic.

Predisposing factors	Enabling factors	Reinforcing factors
Clinician self-efficacy to appropriately determine buprenorphine dosing Clinician self-efficacy to provide patients with follow-up instructions Clinician perceived risk of buprenorphine diversion	Clinician X-waiver to prescribe buprenorphine Process for timing and location of clinician referral to community-based clinic Process for scheduling follow-up appointment at community-based clinic Patient ability to pay for buprenorphine prescription	Extrinsic motivation to obtain/maintain X-waiver

**Table 2:** Intervention alignment to identified factors

Identified factors	Aligned intervention component
Clinician self-efficacy to appropriately determine buprenorphine dosing	Standardized dosing scheme for clinicians
Clinician self-efficacy to provide patients with follow-up instructions	Standardized patient instructions for clinicians
Clinician perceived risk of buprenorphine diversion	Diversion education for clinicians
Clinician X-waiver to prescribe buprenorphine	Funded hybrid trainings for clinicians
Process for timing and location of clinician referral to community-based clinic	Mission, vision, and values document for clinic participation in network
Process for scheduling follow-up appointment at community-based clinic	Centralized referral system
Patient ability to pay for buprenorphine prescription	Patient voucher for buprenorphine prescription
Extrinsic motivation to obtain/maintain X-waiver	Clinician honorariums

**Table 3:** Characteristics of organizations participating in the New York MATTERS^*^ network

Type or organization	Number of organizations
Hospitals	--
Teaching community	10
Nonteaching community	24
Federal government	1
Community-based MOUD clinics	--
Single site	31
Multisite: 2–4 locations	14
Multisite: 5+ locations	2
Retail community pharmacies	--
Local/independent	20
Chain	2

* *MATTERS*, Medication Treatment and Electronic Referrals network; *MOUD*, medications for opioid use disorder.
